# Pattern of drug use among preterm neonates: results from an Italian neonatal intensive care unit

**DOI:** 10.1186/s13052-017-0354-z

**Published:** 2017-04-17

**Authors:** A. Girardi, S. Galletti, E. Raschi, A. Koci, E. Poluzzi, G. Faldella, F. De Ponti

**Affiliations:** 1 Department of Medical and Surgical Sciences, University of Bologna 40138, Bologna, Italy; 2 Present Address: Department of Medical and Surgical Sciences, University of Bologna, via Irnerio 48 40126, Bologna, Italy

**Keywords:** Neonatal intensive care unit, Preterm neonates, Drug use, Nephrotoxicity

## Abstract

**Background:**

Drug use in preterm neonates admitted to Neonatal Intensive Care Unit (NICU) has been investigated, so far, in terms of unauthorized or off-label use; very little is known on the use of combinations of different active substances, which is frequently required in this population (prophylaxis of infections, treatment of concomitant diseases). The aim of this study was to describe the most common patterns of drug use in an Italian NICU, focusing on those with nephrotoxic potential.

**Methods:**

Medical records of preterm neonates (<37 weeks of gestational age) weighing less than 1,500 g at birth and admitted to an Italian NICU were scrutinized in a 3-year retrospective investigation. Analysis included drug exposure, duration of therapies, co-administration of drugs with potential renal side effects; also daily protein supplement was calculated from parenteral nutrition.

**Results:**

A cohort of 159 preterm neonates was selected; 68 were born weighing less than 1,000 g (extremely low birth weight infants, ELBW, Group A), 91 weighed between 1,000 and 1,500 g at birth (Group B). Compared to Group B, neonates of Group A were more likely to receive pharmacological treatments: the most used drugs were antibiotics (especially ampicillin and amikacin, *p =* .07 and *p <* .001, respectively), antifungals (especially fluconazole, *p <* .001), and diuretics (especially furosemide, *p <* .001). Analysis of co-administration of drugs with potential nephrotoxicity showed ampicillin and amikacin as the most reported combination (94.1% of Group A and 31.9% of Group B), the combination of furosemide with antibacterials (ampicillin or amikacin) was also frequently reported, with average period of combination shorter than 2 days.

**Conclusions:**

ELBW infants were exposed to a higher number of drugs compared to other neonates and were more likely to receive associations of drugs with nephrotoxic potential (e.g. furosemide and amikacin), though only for short cycles. Further studies should evaluate the safety profile (especially potential renal side effects) related to most commonly used combinations.

## Background

Neonates, especially those born prematurely, are characterized by several specific pathological conditions and require the administration of several concomitant pharmacological treatments. For instance, in the first postnatal period, neonatologists often have to manage respiratory distress syndrome, patent ductus arteriosus (PDA), necrotizing enterocolitis (NEC) and other infections [1]. At present, very little is known on the actual use of combination of drugs in neonatal intensive care settings and their safety; studies investigating drug use in the neonatal population usually focused on single active substances and their status in terms of unauthorized or off-label use [2–5]. The evidence gathered so far however highlighted increased susceptibility of babies to drug-related toxicity (especially, renal damage [2]).

The issue of evidence-based pharmacological treatment among neonates is unsolved, especially because of known difficulties in performing clinical trials in this population. Also observational research by collecting “real-world” data from Neonatal Intensive Care Units (NICUs) is challenging because of potential heterogeneity of patients enrolled in multicenter studies. Almost all medications are actually used off-label in newborns (especially if preterm); some exceptions are represented by some antibacterials, for instance amikacin. Therefore, National and Regional guidelines (in Italy, as well as in most Western Countries, e.g. British National Formulary for Children) have been created on the basis of consolidated clinical use of off-label drugs in pediatrics, providing information for neonatal and pediatric units. Moreover, the Paediatric Committee of the European Medicines Agency periodically identifies a list of active substances for which data on efficacy and side effects in the pediatric population (including neonates) are requested [6].

Thus, evidence from clinical practice is particularly useful not only for the assessment of the risk-benefit profile of drugs in neonates, but also for the opportunity to add recommendations in this population and to gain insight into relevant unmet clinical needs.

The present study aims at: (1) describing the use of drugs among preterm neonates, especially in terms of co-administrations, and (2) focusing on the use of agents with nephrotoxic potential.

## Methods

### Study Cohort

The study was conducted retrospectively in the tertiary-level NICU of the “S. Orsola-Malpighi” Hospital in Bologna (Northern Italy) after notification to the Institutional Ethics Committee (Comitato Etico Policlinico S.Orsola-Malpighi, 34/2015/U/Oss). Newborns were included in the study on the basis of the following criteria: born at the “S. Orsola-Malpighi” Hospital between January 01, 2009 and December 31, 2011 and admitted to the NICU of the same hospital; gestational age < 37 weeks and weight at birth ≤ 1,500 g. Patients who died within the first 48 h after birth were excluded.

### Data collection

For each neonate, medical records were scrutinized to collect data on twin birth, pathological conditions at birth and during the hospitalization, duration of hospitalization, type of drug prescribed classified according to the anatomical therapeutic chemical (ATC) system (WHO Collaborating Centre For Drug Statistics Methodology, Guidelines for ATC classification and DDD assignment 2009. Oslo, 2008), the starting age and the duration of therapy. Active substances with potential renal side effects were identified according to published data [2]: antibacterials (ampicillin, piperacillin, vancomycin, amikacin), antifungals (amphotericin B), loop diuretics (furosemide), non-steroidal anti-inflammatory drugs (indomethacin, ibuprofen), and paracetamol (acetaminophen). All data acquired from medical records were stored in an electronic database.

### Statistical analysis

Neonates were classified into two groups according to birth weight: Group A neonates weighing ≤ 1,000 g at birth (extremely low birth weight, ELBW) and Group B neonates weighing >1,000 g and ≤1,500 g at birth.

**Fig. 1:**
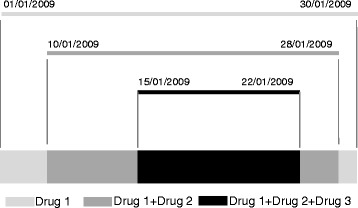
Schematic representation of our approach to describe co-administrations of drugs: for instance, when a second drug was added to drug1, we recorded this exposure as drug1 + drug2 and considered the relevant co-administration time-period

Analysis of drug use was performed with Access® software and included: (a) exposure, defined as the number of unique active substances reported for each neonate, (b) courses, defined as the number of times a unique active substance was reported for a single patient with a specific start date (the analysis of courses shows if a pharmacological treatment was chosen more than once during hospitalization), (c) courses per exposure (number of times neonates were exposed to more than one course of a specific drug) and (d) duration *per courses* (when a unique active substance was administered more than once for a single patient, exposure time for each course was calculated). Co-administrations of drugs were calculated on the basis of each neonate daily therapy and assembling together treatments used in the same period of time (Fig. 1). For the estimation of protein intake, we recorded the daily amount and duration of protein administration included in the parenteral nutrition. Differences between groups were evaluated using the Chi-square test and Fisher’s exact test; statistical significance was defined for *p* ≤ 0.05.

## Results

Among all preterm neonates admitted to NICU in the study period, medical records were available for 159 patients: 68 neonates weighing less than 1,000 g at birth (Group A), and 91 neonates weighing > 1,000 g and ≤1,500 g (Group B). Characteristics of the two populations are presented in Table 1. All neonates of Group A needed pharmacological treatments and they received a higher number of different active substances compared to Group B (Table 1): 95,6% of Group A and 37,4% of Group B received more than 10 different drugs throughout their stay in NICU; moreover, all ELBW infants and 93,4% of Group B were exposed to associations.

**Table 1:** Characteristics of the study population and pharmacological treatment

	GROUP A	GROUP B
	*N =* 68	*N =* 91
Gesational age, weeks		
Average	26	30
Range	22 – 32	27 – 36
Birth weight, g		
Average	739	1309
Range	380 – 1000	1023 – 1532
Discharge age, days		
Average	57	23
Range	2 – 218	1 – 175
Singleton birth, %	77.9	71.4
Outcome, %		
Death	17.7	3.3
Transfer	4.4	5.5
Home	77.9	91.2
Diseases, %		
Respiratory distress syndrome	82.4	86.8
Anemia	75.0	35.2
Hyperbilirubinemia	70.6	93.4
Patent ductus arteriosus	52.9	42.9
Sepsis	38.2	11.0
Hyaline membrane disease	27.9	5.5
Intrauterine growth restriction	23.5	17.6
Central nervous system impairment	22.1	8.8
Necrotizing enterocolitis	13.2	5.5
Cardiac malformation	11.8	15.4
Pharmacological treatments, %	100	95.7
Number of drugs, %		
≤ 5	0	17.6
5–10	4.4	40.7
11–20	51.5	33.0
> 20	44.1	4.4
Combination of drugs, %	100	93.4
Route of administration, %		
Drugs for systemic use	100	96.7
Drugs for topical use	82.4	57.1
Drugs for ophthalmic use	44.1	14.3
Exposure to at least one drug with potential renal toxicity^a^, %	100	93.4

GROUP A: birth weight ≤ 1000 g; GROUP B: birth weight >1000 g and ≤1500 g; ^a^according to available data [2]

All neonates of Group A and 93.4% of Group B were exposed to at least one drug with potential renal side effect. At birth, all neonates received a single administration of ophthalmic antibiotic (tobramycin) and vitamin K; frequency of exposure to drugs, duration and courses of pharmacological treatments in both groups are shown in Table 2 and Table 3. In the first days of life, neonates were especially exposed to antibacterials, in particular ampicillin and amikacin, and antifungal agents, mainly fluconazole. In case another antibacterial was needed after the end of early treatment with ampicillin or amikacin, the most prescribed active substances in both groups were piperacillin, vancomycin, clarithromycin and erythromycin, and they were started on average three weeks after birth. Neonates weighing less than 1,500 g at birth were more likely to receive antibacterials (with the exception of ampicillin) and antifungals compared to Group B (Table 4); moreover, in neonates of Group A, treatment with anti-infective agents lasted more than three times as long as in Group B.

**Table 2:** Most commonly reported active substances in Group A

Active substance	Exposure (*N =* 68)	% (tot. 100)	Courses	Courses/exposure	Duration *per course*, d (average)
Ampicillin^a^	66	97.1	66	-	-
Amikacin^a^	66	97.1	76	1.2	6
Caffeine	65	95.6	76	1.2	35
Fluconazole	63	92.6	83	1.3	25
Calcitriol	60	88.2	74	1.2	39
Furosemide^a^	49	72.1	106	2.2	6
Fentanyl	45	66.2	84	1.9	8
Hydrochlorothiazide	34	50.0	50	1.5	31
Spironolactone	34	50.0	51	1.5	30
Piperacillin and enzyme inhibitor^a^	32	47.1	43	1.3	9
Lung surfactant - natural phospholipids	31	45.6	34	1.1	3
Dopamine	30	44.1	38	1.3	9
Metronidazole	27	39.7	30	1.1	11
Calcium folinate	26	38.2	27	1.0	35
Tobramycin	26	38.2	35	1.3	6
Folic acid	25	36.8	28	1.1	31
Dobutamine	25	36.8	32	1.6	6
Immunoglobulins, normal human, for intravascular administration	24	35.3	30	1.3	3
Vancomycin^a^	24	35.3	37	1.3	9
Heparinoids for topical use	23	33.8	30	1.5	4
Ibuprofen^a^	22	32.4	22	-	-
Ranitidine	21	30.9	22	1.1	24
Filgrastim	21	30.9	26	1.2	1
Clarithromycin	20	29.4	24	1.2	13
Dexamethasone	19	27.9	36	1.9	12
Calcifediol	18	26.5	22	1.2	32
Atropine	17	25.0	19	1.1	1
Albumin	16	23.5	23	1.4	1
Betamethasone	14	20.6	17	1.2	6
Erythromycin ethylsuccinate	13	19.1	13	-	-
Lorazepam	12	17.6	23	1.9	1
Doxapram	12	17.6	15	1.3	12
Phytomenadione	11	16.2	13	1.2	17
Oxacillin	11	16.2	13	1.2	8
Paracetamol (Acetaminophen)^a^	11	16.2	19	1.7	6
Beclometasone	11	16.2	13	1.1	11
Ferrous sulfate	10	14.7	11	1.1	16
Amphotericin B^a^	9	13.2	10	1.6	18
Midazolam	9	13.2	14	1.1	10
Insulin (human)	9	13.2	10	1.6	3
Antacids with sodium bicarbonate	7	10.3	11	1.3	4
Glyceryl trinitrate	7	10.3	7	-	-
Phenobarbital	6	8.8	6	-	-
Ceftazidime	6	8.8	9	1.5	14
Mupirocin	6	8.8	8	1.3	4
Morphine	5	7.4	7	1.4	20
Hydrocortisone	4	5.9	5	1.3	8
Calcium levofolinate	4	5.9	4	-	-
Erythromycin	4	5.9	6	1.5	14
Naloxone	4	5.9	4	-	-
Epinephrine	3	4.4	3	-	-
Indometacin^a^	3	4.4	3	-	-
Epoprostenol	3	4.4	6	2.0	5
Linezolid	3	4.4	3	-	-

Birth weight ≤ 1000 g; frequency > 2; ^a^drugs with potential renal side effects [2]

**Table 3:** Most commonly reported active substances in Group B

Active substance	Exposure (*N =* 91)	% (100)	Courses	Courses/exposure	Duration *per course*, d (average)
Ampicillin^a^	78	85.7	79	1.0	5
Caffeine	69	75.8	70	1.0	26
Calcitriol	61	67.0	65	1.1	19
Fluconazole	45	49.5	50	1.1	16
Lung surfactant - natural phospholipids	34	37.4	34	-	-
Fentanyl	32	35.2	36	1.1	7
Amikacin^a^	32	35.2	33	1.0	4
Calcifediol	25	27.5	27	1.1	18
Furosemide^a^	23	25.3	32	1.4	8
Atropine	22	24.2	22	-	-
Folic acid	16	17.6	18	1.1	11
Calcium folinate	13	14.3	14	1.1	15
Vancomycin^a^	13	14.3	16	1.2	10
Heparinoids for topical use	12	13.2	12	-	-
Piperacillin and enzyme inhibitor^a^	11	12.1	13	1.2	10
Dopamine	11	12.1	13	1.2	6
Ibuprofen^a^	11	12.1	11	-	-
Filgrastim	10	11.0	11	1.1	1
Tobramycin	9	9.9	10	1.1	4
Ranitidine	8	8.8	10	1.3	21
Immunoglobulins, normal human, for intravascular administration	8	8.8	8	-	-
Hydrochlorothiazide	8	8.8	8	-	-
Spironolactone	8	8.8	8	-	-
Lorazepam	8	8.8	15	1.9	3
Paracetamol (Acetaminophen)^a^	7	7.7	12	1.7	4
Metronidazole	7	7.7	8	1.1	8
Ferrous sulfate	7	7.7	7	-	-
Piperacillin^a^	7	7.7	7	-	-
Doxapram	6	6.6	8	1.3	11
Ceftazidime	6	6.6	8	1.3	11
Mupirocin	6	6.6	6	-	-
Alginic acid	6	6.6	8	1.3	12
Glyceryl trinitrate	5	5.5	5	-	-
Claritromycin	5	5.5	5	-	-
Dobutamine	5	5.5	6	1.2	9
Naloxone	4	4.4	4	-	-
Midazolam	4	4.4	5	1.3	5
Betamethasone	3	3.3	6	2.0	11
Phytomenadione	3	3.3	3	-	-
Beclometasone	3	3.3	4	1.3	3
Captopril	3	3.3	6	2.0	11
Erythromycin	3	3.3	4	1.3	10

Birth weight >1000 g and ≤1500 g; frequency > 2; ^a^drugs with potential renal side effects [2]

**Table 4:** Differences between active substances use among groups for the main drug classes

	GROUP A % (*N =* 68)	GROUP B % (*N =* 91)	*p*	GROUP A overall exposure, d	GROUP B overall exposure, d	*p*
ANTIBACTERIALS FOR SYSTEMIC USE	100,0	91.2	.07	48	14	
Ampicillin^a^	97.1	87.5	.07	7	5	.16
Amikacin^a^	97.1	35.2	<.0001	7	4	.33
Piperacillin and enzyme inhibitor^a^	47.1	12.1	<.0001	13	11	.03
Metronidazole	39.7	7.7	<.0001	13	9	.10
Vancomycin^a^	35.3	14.3	<.0001	13	13	.01
Clarithromycin	29.4	5.5	<.0001	15	13	.02
Erythromycin ethylsuccinate	19.1	2.2	<.0001	15	8	.25
ANTIMYCOTICS FOR SYSTEMIC USE	92.6	49.5	<.0001	36	18	
Fluconazole	92.6	49.5	<.0001	32	18	.78
Amphotericin B^a^	13.2	1.1	.03	20	1	.01
RESPIRATORY SYSTEM PRODUCTS	55.9	39.6	.02	7	3	
Lung surfactant - natural phospholipidis	45.6	37.4	.69	3	1	.85
Doxapram	17.6	6.6	.21	15	14	.32
Caffeine	95.6	75.8	.02	45	27	n.a.
DIURETICS	83.8	31.9	<.0001	65	26	
Furosemide^a^	72.1	25.3	.77	12	11	.08
Hydrochlorothiazide	50,0	8.8	.07	46	26	.31
Spironolactone	50,0	8.8	.07	46	26	.31
CARDIAC THERAPY	55.9	28.6	<.0001	23	9	
Dopamine	44.1	12.1	.10	12	7	.52
Dobutamine	36.8	5.5	.02	7	11	.02
Ibuprofen^a^	32.4	12.1	.43	2	2	.37

GROUP A: birth weight ≤ 1000 g; GROUP B: birth weight > 1000 g and ≤1500 g; ^a^drugs with potential renal side effects [2]

Caffeine was widely used, especially among neonates of Group A compared to Group B, as well as lung surfactants, whose main indications are prevention and treatment of respiratory distress syndrome; only for few cases, the additional administration of doxapram was requested. Neonates of Group A were more likely to receive diuretics compared to Group B; moreover, neonates of Group A treated with furosemide received at least two different administrations, with an average exposure duration of 12 days.

Almost all neonates of Group A were exposed to a combination of drugs with nephrotoxic potential; the most commonly reported combination in both groups was ampicillin and amikacin (94.1% Group A and 31.9% Group B), also the association of furosemide with ampicillin or amikacin was frequently reported; the average period of co-administration did not exceed 2 days, with the exception of piperacillin and vancomycin in Group B (Table 5). Notably, some of the investigated combinations, such as ibuprofen with amikacin, and indomethacin with amikacin, were not prescribed.

**Table 5:** Most commonly reported associations of drugs with potential renal side effects [2]

		GROUP A		GROUP B
	% (*N =* 68)	Duration *per course*, average	% (*N =* 91)	Duration *per course*, average
Ampicillin, Amikacin	94,1	1,5	31,9	1,4
Piperacillin, Vancomycin	32,4	1,9	9,9	2,5
Amikacin, Furosemide	20,6	1,6	7,7	1,3
Ampicillin, Furosemide	19,1	1,1	15,4	1,6
Amikacin, Acetaminophen	5,9	1,5	2,2	1,2
Amikacin, Amphotericin B	2,9	1,7	-	-

GROUP A: birth weight ≤ 1000 g; GROUP B: birth weight >1000 g and ≤1500 g

Parenteral nutrition were enriched with proteins in 67/69 cases of Group A and 83/91 cases of Group B; no significant differences were shown for dosages, whereas the overall exposure period in Group A was twice as long as in Group B (Table 6).

**Table 6:** Characteristics of protein administration (as part of parenteral nutrition) among groups

	GROUP A (*N =* 69)	GROUP B (*N =* 91)	*p*
Patients receiving protein supplement, %	98.5	91.2	
Average dosage, g/kg	2.36	2.11	.83
min dosage, g/kg	0.5	0.5	
max dosage, g/kg	3.75	3.5	
Overall exposure (on average), days	35	17	.01
min, days	1	1	
max, days	111	61	
Combination with drugs, %			
ampicillin, amikacin	85.3	33.3	<.0001
ampicillin	66.2	80.8	.97
furosemide	44.1	11.5	<.0001
piperacillin	29.4	16.7	.03
piperacillin, vancomycin	26.5	7.7	<.0001

GROUP A: birth weight ≤ 1000 g; GROUP B: birth weight >1000 g and ≤1500 g

## Discussion

### Drug use and combination

In the present study, we described the current medication use in an Italian NICU and the combination of drugs: almost all neonates admitted to NICU needed a combination of drugs, especially ELBW neonates. Combination of drugs with potential nephrotoxicity regarded antibacterials and furosemide, and their combination did not exceed 2.5 days.

Most preterm newborns received more than 10 drugs during their stay in NICU, with large differences between ELBW and the others. The most commonly used drugs were antimicrobials, especially ampicillin and amikacin, which were usually co-administered in ELBW for prophylactic purposes starting from the first postnatal day. Also furosemide was frequently used, usually starting later, in case of specific cardio-vascular impairment. For all these three medications, potential nephrotoxicity is well known. Apart from ampicillin and amikacin, also combinations with caffeine, used to prevent apnea, and with fluconazole were frequently found.

The high number of different pharmacological treatments used among preterm neonates in the present investigation is likely to be related to a dual need: to preserve vital status of those particularly frail babies through preventive care therapies, and to treat specific pathological conditions.

The higher number of drugs received by ELBW is driven by the fact that these patients are more likely to suffer from concomitant diseases (especially sepsis) requiring intensive prophylaxis. Prevention of neonatal infections is a clinical priority for neonatologists, as recognized in local protocols, because early and late onset neonatal sepsis are identified as a major cause of mortality and are correlated to neurodevelopmental impairment in the first years of life [7, 8], again especially in ELBW [9]. In the present study, apart from the use of specific antimicrobials early after birth, the use of other antibacterials during hospitalization was frequent, meaning a high incidence of suspected late-onset infections (e.g., almost a half of ELBW received piperacillin). Metronidazole should be separately discussed because of its main indication in NEC treatment; as a consequence, ELBW patients were more exposed to metronidazole as they were more likely to suffer from this pathological condition.

Our results on most used classes of drugs (antimicrobials, cardiovascular agents, analgesics and respiratory drugs) are in accordance to other studies investigating the profile of drug use in NICUs, performed in other Western Countries [5, 10–12].

As regards antibiotic choice, our findings are comparable to other Italian NICUs [3, 13], but differ from other Countries [10, 11, 14, 15]. Inter-Country and inter-centre variability in antibiotic choice, dose regimen and intervals in NICUs is commonly reported worldwide [16–18] and a number of factors may explain this difference: (a) the lack of clinical trials performed in this population resulted in deficiency of international guidelines, (b) clinician’s attitude and hospital policy may also play an important role in both the choice of active substances and the pattern of antibiotic use, (c) moreover, the local epidemiology of bacterial infection is an essential factor, as well as previous maternal infections.

While the choice of antibacterial agents is still debated, the use of fluconazole among newborns as antifungal prophylaxis is widely acknowledged because of its general safety and proven efficacy for the prevention of invasive candidiasis [19–22].

### Drugs with potential renal side effects

Renal damage onset, particularly acute kidney injury, is common among preterm neonates and correlates with high mortality rate [23, 24]. Among factors that can mitigate this risk, the short-term administration of drugs with nephrotoxic potential, such as aminoglycosides and diuretics, is recognized [25, 26].

Moreover, recent evidence on aminoglycoside use in preterm neonates and kidney damage shows that amikacin seems to be safer than gentamicin [27].

Our findings on combination of drugs with potential nephrotoxicity showed that the combinations of two different antibacterial agents and an antibacterial with furosemide were frequently reported in preterm neonates, though for short periods. The benefit-risk profile of combination of drugs in preterm neonates remains almost unexplored. To the best of our knowledge, only one study assessed the clinical consequences of the use of one aminoglycoside and furosemide in combination in the neonatal intensive care setting, showing that cycles longer than 4.5 days were associated with increased risk of acute kidney injury [28]. In our population, this combination did not exceed 2 days, thus minimizing this concern.

Factors that can aggravate kidney damage are prematurity, diet and concomitant disorders, for instance, perinatal asphyxia, respiratory distress syndrome and sepsis [29–32], whereas the effect of protein intake on renal function in the preterm population is still to be clearly characterized. Nutritional supply is essential for the prevention of growth failure of premature babies: insufficient energy and macronutrients intake may lead to unbalanced growth, altered neurological development and increased risk of morbidity [33].

Some limitations of this study should be acknowledged to better interpret our findings: the study was conducted in a single Italian university hospital, which may limit the generalizability of our findings to other settings; also, the amount of protein intake here described did not take into account enteral nutrition, resulting in underestimation of the total amount of protein intake.

## Conclusions

With this retrospective study we presented an accurate description of pattern of drug use in an Italian NICU and, by using a novel approach, we further described the combination of active substances. Neonates born prematurely, especially ELBW, received a number of different pharmacological treatments from the first day after birth and in several cases drugs were administered in combination. Most of the drugs used in combination have potential renal side effects (e.g. amikacin and ampicillin), but they were administered for short periods. For most of those drugs, the risk-benefit profile is still not fully assessed in the neonatal population, and scanty evidence is available for their use in combination. Further studies, involving more than one Centre, should explore the safety of the most used combinations of drugs in NICU patients, such as aminoglycosides and furosemide, with special attention to renal toxicity.

## Abbreviations

ELBW: Extremely low birth weight
NEC: Necrotizing enterocolitis
NICU: Neonatal intensive care unit
